# Redundant Information Neural Estimation

**DOI:** 10.3390/e23070922

**Published:** 2021-07-20

**Authors:** Michael Kleinman, Alessandro Achille, Stefano Soatto, Jonathan C. Kao

**Affiliations:** 1 Department of Electrical and Computer Engineering, University of California, Los Angeles, CA 90095, USA; kao@seas.ucla.edu; 2 Department of Computational and Mathematical Sciences, Caltech, Pasadena, CA 91125, USA; aachille@caltech.edu; 3 Department of Computer Science, University of California, Los Angeles, CA 90095, USA; soatto@cs.ucla.edu

**Keywords:** redundant information, usable information, Partial Information Decomposition

## Abstract

We introduce the Redundant Information Neural Estimator (RINE), a method that allows efficient estimation for the component of information about a target variable that is common to a set of sources, known as the “redundant information”. We show that existing definitions of the redundant information can be recast in terms of an optimization over a family of functions. In contrast to previous information decompositions, which can only be evaluated for discrete variables over small alphabets, we show that optimizing over functions enables the approximation of the redundant information for high-dimensional and continuous predictors. We demonstrate this on high-dimensional image classification and motor-neuroscience tasks.

## 1. Introduction

Given a set of sources $X_{1},\ldots ,X_{n}$ and a target variable *Y*, we study how information about the target *Y* is distributed among the sources: different sources may contain information that no other source has (“unique information”), contain information that is common to other sources (“redundant information”), or contain complementary information that is only accessible when considered jointly with other sources (“synergistic information”). Such a decomposition of the information across the sources can inform the design of multi-sensor systems (e.g., to reduce redundancy between sensors), or support research in neuroscience, where neural activity is recorded from two areas during a behavior. For example, a detailed understanding of the role and relationship between brain areas during a task requires understanding how much unique information about the behavior is provided by each area that is not available to the other area, how much information is redundant (or common) to both areas, and how much additional information is present when considering the brain areas jointly (i.e., information about the behavior that is not available when considering each area independently).

**Figure A1:**
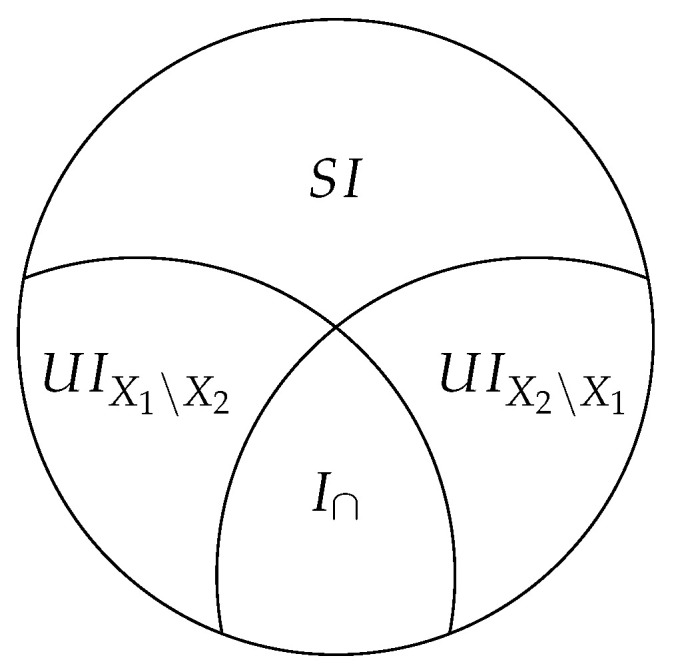
Decomposition of the mutual information of a sources $X_{1},X_{2}$ and target *Y* into the synergistic information SI, the unique information UI of $X_{1}$ with respect to *Y* and $X_{2}$ with respect to *Y*, and the redundant information $I_{\cap }$. Figure adapted from [6].

Standard information–theoretic quantities conflate these notions of information. Williams and Beer [1] therefore proposed the Partial Information Decomposition (PID), which provides a principled framework for decomposing how the information about a target variable is distributed among a set of sources. For example, for two sources $X_{1}$ and $X_{2}$, the PID is given by

(1) $$I\left(X_{1},X_{2};Y\right)=UI\left(X_{1};Y\right)+SI+UI\left(X_{2};Y\right)+I_{\cap },$$

where UI represents the “unique” information, SI the “synergistic” information, and $I_{\cap }$ represents the redundant information, shown in Figure A1. We provide details in Appendix C.1, describing how standard information–theoretic quantities, such as the mutual information $I(X_{1};Y)$ and conditional mutual information $I(X_{2};Y|X_{1})$, are decomposed in terms of the PID constituents.

Despite efforts and proposals for defining the constituents [2,3,4,5,6,7], existing definitions involve difficult optimization problems and remain only feasible in low-dimensional spaces, limiting their practical applications. One way to sidestep these difficult optimization problems is to assume a joint Gaussian distribution over the observations [8], and this approach has been applied to real-world problems [9]. To enable optimization for high-dimensional problems with arbitrary distributions, we reformulate the redundant information through a variational optimization problem over a restricted family of functions. We show that our formulation generalizes existing notions of redundant information. Additionally, we show that it correctly computes the redundant information on canonical low-dimensional examples and demonstrate that it can be used to compute the redundant information between different sources in a higher-dimensional image classification and motor-neuroscience task. Importantly, RINE is computed using samples from an underlying distribution, which does not need to be known.

Through RINE, we introduce a similarity metric between sources which is task dependent, applicable to continuous or discrete sources, invariant to reparametrizations, and invariant to addition of extraneous or noisy data.

## 2. Related Work

Central to the PID is the notion of redundant information $I_{\cap }$, and much of the work surrounding the PID has focused on specifying the desirable properties that a notion of redundancy should follow. Although there has been some disagreement as to which properties a notion of redundancy should follow [1,4,7], the following properties are widely accepted:

- Symmetry: $I_{\cap }\left(X_{1};\ldots ;X_{n}\;\to \;Y\right)$ is invariant to the permutation of $X_{1},\ldots ,X_{n}$.
- Self-redundancy: $I_{\cap }\left(X_{1}\;\to \;Y\right)=I\left(X_{1};Y\right)$.
- Monotonicity: $I_{\cap }\left(X_{1};\ldots ;X_{n}\;\to \;Y\right)\leq I_{\cap }\left(X_{1};\ldots ;X_{n-1}\;\to \;Y\right)$.

Several notions of redundancy have been proposed that satisfy these requirements, although we emphasize that these notions were generally not defined with efficient computation in mind.

Griffith et al. [2] proposed a redundancy measure $I_{\cap }^{\wedge }$, defined through the optimization problem:

(2) $$I_{\cap }^{\wedge }\left(X_{1};\ldots ;X_{n}\;\to \;Y\right):=\max_{Q}\;I\left(Y;Q\right)\;s.t.\;\forall i\;\exists f_{i}\;Q=f_{i}\left(X_{i}\right)$$

where *Q* is a random variable and $f_{i}$ is a deterministic function. The redundant information is thus defined as the maximum information that a random variable *Q*, which is a deterministic function of all $X_{i}$, has about *Y*. This means that *Q* captures a component of information common to the sources $X_{i}$.

An alternative notion of redundant information $I_{\cap }^{\mathrm{GH}}$ [5,10] with a less restrictive constraint is defined in terms of the following optimization problem:

(3) $$I_{\cap }^{\mathrm{GH}}\left(X_{1};\ldots ;X_{n}\;\to \;Y\right):=\max_{Q}I\left(Y;Q\right)\;s.t.\;\forall i\;I\left(Y;Q|X_{i}\right)=0.$$

$I_{\cap }^{\mathrm{GH}}$ reflects the maximum information between *Y* and a random variable *Q* such that $Y-X_{i}-Q$ forms a Markov chain for all $X_{i}$, relaxing the constraint that *Q* needs to be a deterministic function of $X_{i}$.

We show in Section 3 that our definition of redundant information is a generalization of $I_{\cap }^{\wedge }$ and can be extended to compute $I_{\cap }^{\mathrm{GH}}$.

The main hurdle in applying these notions of information to practical problems is the difficulty of optimizing over all possible random variables *Q* in a high-dimensional setting. Moreover, even if that was possible, such unconstrained optimization could recover degenerate forms of redundant information that may not be readily “accessible” to any realistic decoder. In the next section we address both concerns by moving from the notion of Shannon Information to the more general notion of Usable Information [11,12,13].

###  Usable Information in a Random Variable

An orthogonal line of recent work has looked at defining and computing the “usable” information $I_{u}\left(X;Y\right)$ that a random variable *X* has about *Y* [11,12,13]. This aims to capture the fact that not all information contained in a signal can be used for inference by a restricted family of functions. Given a family of decoders V⊆U={f:X→P(Y)}, the usable information that *X* has about *Y* is defined as

(4) $$I_{u}\left(X;Y\right)=H\left(Y\right)-H_{V}\left(Y|X\right),$$

where $H_{V}\left(Y|X\right)$ is defined as

(5) $$H_{V}\left(Y|X\right)=\underset{f\in V}{\inf}E_{x,y∼X,Y}\left[-\log f(y|x)\right].$$

Thus, the “usable” information differs from Shannon’s mutual information in that it involves learning a decoder function *f* in a model family V, which is a subset of all possible decoders U. When the “usable” information is defined such that the model family corresponds to the universal model family, the definition recovers Shannon’s mutual information, $I\left(X;Y\right)=H\left(Y\right)-H_{U}\left(Y|X\right)$. However, in many cases, the “usable information” is closer to our intuitive notion of information, reflecting the amount of information that a learned decoder, as opposed to the optimal decoder, can extract under computational constraints [11]. We extend these ideas to compute the “usable redundant information” in the next section.

## 3. Redundant Information Neural Estimator

We introduce the Redundant Information Neural Estimator (RINE), a method that enables the approximation of the redundant information that high-dimensional sources contain about a target variable. In addition to being central for the PID, the redundant information also has direct applicability in that it provides a task-dependent similarity metric that is robust to noise and extraneous input, as we later show in Section 4.4.

Our approximation leverages the insight that existing definitions of redundancy can be recast in terms of a more general optimization over a family of functions, similar to how the “usable information” was defined above. To this end, given two sources, we define a notion of redundancy, RINE, through the following optimization over models $f_{1},f_{2}\in V\subseteq U=\left\{f:X\to P\left(Y\right)\right\}$.

(6) $$\begin{array}{cc} L_{\cap }^{V}\left(X_{1};X_{2}\;\to \;Y\right):= & \min_{f_{1},f_{2}\in V}\frac{1}{2}\left[H_{f_{1}}\left(Y|X_{1}\right)+H_{f_{2}}\left(Y|X_{2}\right)\right] \end{array}$$

(7) $$\begin{array}{cc} \; & \;\;\;s.t.\;\;D(f_{1},f_{2})=0 \end{array}$$

(8) $$\begin{array}{cc} I_{\cap }^{V}\left(X_{1};X_{2}\;\to \;Y\right):= & H\left(Y\right)-L_{\cap }^{V}, \end{array}$$

where $H_{f_{i}}\left(Y|X_{i}\right)$ denotes the cross-entropy when predicting *Y* using the decoder $f_{i}\left(y|x\right)$ and $D\left(f_{1},f_{2}\right)=E_{x_{1},x_{2}}\left[∥f_{1}\left(y|x_{1}\right)-f_{2}\left(y|x_{2}\right){\}∥}_{1}\right]$ denotes the expected difference of the predictions of the two decoders. Importantly, the model family V can be parametrized by neural networks, enabling optimization over the two model families with backpropagation. In general, one can optimize over different model families $V_{1}$ and $V_{2}$, but for notational simplicity we assume we optimize over the same model family V in the paper. Note that here we constrained the predictions directly, as opposed to using an intermediate random variable *Q*. In contrast, direct optimization of Equations (2) and (3) is only feasible for discrete sources with small alphabets [7]. Our formulation can be naturally extended to *n* sources (Appendix C.8) and other divergence measures between decoders. Since our formulation involves learning decoders that map the sources to target predictions, the learned decoder can safely ignore task-irrelevant variability, such as noise, as we demonstrate in Section 4.4.

To solve the constrained minimization problem in Equations (6) and (7), we can minimize the corresponding Lagrangian:

(9) $$L_{\cap }^{V}\left(X_{1};X_{2}\;\to \;Y,\beta \right):=\min_{f_{1},f_{2}\in V}\frac{1}{2}\left[H_{f_{1}}\left(Y|X_{1}\right)+H_{f_{2}}\left(Y|X_{2}\right)\right]+\beta D\left(f_{1},f_{2}\right).$$

When β→∞ the solution to the Lagrangian is such that $D(f_{1},f_{2})\to 0$, thus satisfying the constraints of the original problem. In practice, when optimizing this problem with deep networks, we found it useful to start the optimization with a low value of β, and then increase it slowly during training to some sufficiently high value (β=50 in most of our experiments). Note that while H(Y) does not appear in the Lagrangian, it is still used to compute $I_{\cap }^{V}$, as in Equation (8). The Lagrangian is optimized, using *samples* from an underlying distribution $p(X_{1},X_{2},Y)$; importantly, the underlying distribution can be continuous or discrete.

Our definition of V-redundant information (Equation (8)) is a generalization of $I_{\cap }^{\wedge }$ (Section 2) as shown by the following proposition:

**Proposition** **1** (Appendix B). *Let V={f:X→P(Y)} consist of the family of deterministic functions from X to distributions over Y. Then $I_{\cap }^{V}=I_{\cap }^{\wedge }$.*

Our formulation involving a constrained optimization over a family of functions is general: indeed, optimizing over stochastic functions or channels with an appropriate constraint can recover $I_{\cap }^{\mathrm{GH}}$ or $I_{\cap }^{K}$ [7] (described in the Appendix) but the computation in practice becomes more difficult.

Our definition of redundant information is also invariant to reparametrization of the sources as shown by the following proposition:

**Proposition** **2** (Appendix B). *Let t:X→X be any invertible transformation in V. Then,*

(10) $$I_{\cap }^{V}\left(X_{1};X_{2}\;\to \;Y\right)=I_{\cap }^{V}\left(t_{1}\left(X_{1}\right);t_{2}\left(X_{2}\right)\;\to \;Y\right).$$

Note that when V=U, $I_{\cap }^{V}$ is invariant to *any* invertible transformation. In practice, when optimizing over a subset V⊆U, our definition is invariant to transformations that preserve the usable information (this accounts for practical transformations, for example the reflection or rotation of images). As an example of transformations that lie in V, consider the case in which V is a set of linear decoders. This model family is closed under any linear transformation t(X) applied to the source, since the composition of linear functions is still a linear function.

As an additional example, the family of fully connected networks is closed to permutations of the pixels of an image since there exists a corresponding network f∈V that would behave the same on the transformed image. The family of convolutional networks, for a given architecture on the other hand, is not closed under arbitrary transformations of the pixels, but it is closed, e.g., under rotations/flips of the image.

In contrast, complex transformations such as encryption or decryption (which preserve Shannon’s mutual information) can decrease or increase respectively the usable information content with respect to the model family V. Arguably, such complex transformations do modify the “information content” or the “usable information” (in this case measured with respect to V) even though they do not affect Shannon’s mutual information (which assumes an optimal decoder in U that may not be in V).

### Implementation Details

In our experiments, we optimize over a model family V of deep neural networks, using gradient descent. In general, the model family to optimize over should be selected such that it is not so complicated that it overfits to spurious features of the finite training set, but has high enough capacity to learn the mapping from source to target.

**Figure 1:**
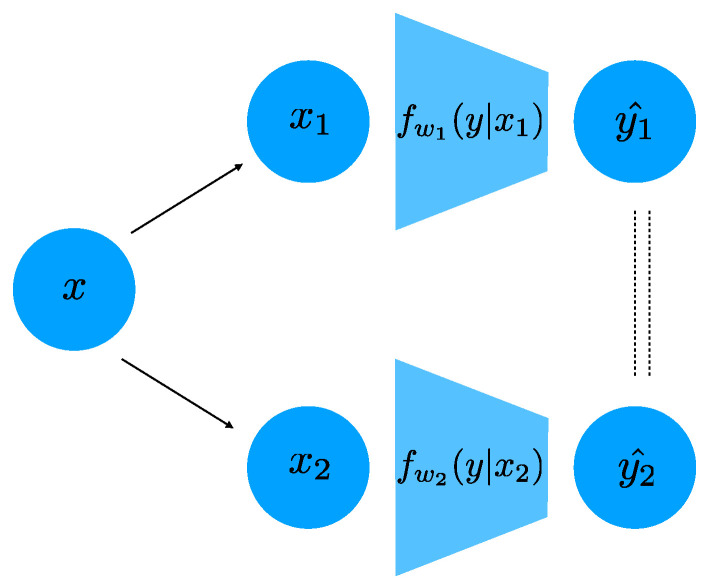
A schematic of our architecture for two sources $X_{1}$ and $X_{2}$. Note that the two networks do *not* share weights. The dashed lines indicate that the predictions are constrained to be similar.

We parametrize the distribution $f_{i}\left(y|x\right)$ in Equation (9), using a deep neural network. In particular, in the case that *y* is discrete (which is the case in all our experiments), the distribution $f_{i}\left(y|x\right)=\mathrm{softmax}\left(h_{w_{i}}\left(x\right)\right)$ is parametrized as the softmax of the output of a deep network with weights $w_{i}$. In this case, the distance $D(f_{1},f_{2})$ can be readily computed as the average $L_{1}$ distance between the softmax outputs of the two networks $h_{w_{1}}\left(x_{1}\right)$ and $h_{w_{2}}\left(x_{2}\right)$ for different inputs $x_{1}$ and $x_{2}$. If the task label *y* is continuous, for example in a regression problem, one can parametrize $f_{i}\left(y|x\right)=N\left(h_{w_{i}}\left(x\right),\sigma^{2}I\right)$ using a Normal distribution whose means is the output of a DNN. We optimize over the weights parametrizing all $f_{i}\left(y|x\right)$ jointly, and we show a schematic of our architecture in Figure 1.

Once we parametrize $f_{1}$ and $f_{2}$, we need to optimize the weights in order to minimize the Lagrangian in Equation (9). We do so using Adam [14] or stochastic gradient descent, depending on the experiment. For images we optimize over ResNet-18’s [15], and for other tasks we optimize over fully-connected networks. The hyperparameter β needs to be high enough to ensure that the constraint is approximately satisfied. However, we found that starting the optimization with a very high value for β can destabilize the training and make the network converge to a trivial solution, where it outputs a constant function (which trivially satisfies the constraint). Instead, we use a reverse-annealing scheme, where we start with a low beta and then slowly increase it during training up to the designated value (Appendix C.3). A similar strategy is also used (albeit in a different context) in optimizing β-VAEs [16].

## 4. Results

We apply our method to estimate the redundant information on canonical examples that were previously used to study the PID, and then demonstrate the ability to compute the redundant information for problems where the predictors are high dimensional.

### 4.1. Canonical Examples

We first describe the results of our method on standard canonical examples that have been previously used to study the PID. They are particularly appealing because for these examples, it is possible to ascertain ground truth values for the decomposition. Additionally, the predictors are low dimensional and have been previously studied, allowing us to compare our variational approximation. We describe the tasks, the values of the sources $X_{1},X_{2}$, and the target *Y* for in Appendix A. Briefly, in the UNQ task, each input $X_{1}$ and $X_{2}$ contributes 1 bit of unique information about the output, and there is no redundant information. In the AND task, the redundant information should be in the interval [0, 0.311] depending on the stringency of the notion of redundancy used [5]. When using deterministic decoders, as we do, we expect the redundant information to be 0 bits (not 0.311 bits). The RDNXOR task corresponds to a redundant XOR task, where there is 1 bit of redundant and 1 bit of synergistic information. Finally, the IMPERFECTRDN task corresponds to the case where $X_{1}$ fully specifies the output, with $X_{2}$ having a small chance of flipping one of the bits. Hence, there should be 0.99 bits of redundant information. As we show in Table 1, RINE (optimizing with a deterministic family; Appendix C.4) recovers the desired values on all these canonical examples.

### 4.2. Redundant Information in Different Views of High-Dimensional Images

To the best of our knowledge, computations of redundant information have been limited to predictors that were one-dimensional [2,5,6,7]. We now show the ability to compute the redundant information when the predictors are high dimensional. We focus on the ability to predict discrete target classes, corresponding to a standard classification setting. In particular, we analyze redundant information between left and right crops of an image (to simulate a system with two stereo cameras), between different color channels of an image (sensors with different frequency bands), and finally between the high and low spatial frequency components of an image.

**Figure 2:**
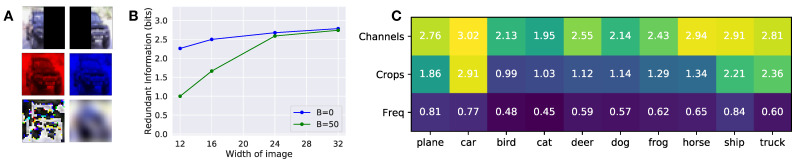
(**A**) Examples of the different views of the image used in the experiment. (**B**) Redundant information of different crops of CIFAR-10 images. Redundant information as a function of the width of each partition, for different values of β. A width of 16 means that both $X_{1}$ and $X_{2}$ is a 16 × 32 image. The images begin from opposing sides, so in the case of the 16 × 32 image, there is no overlap between $X_{1}$ and $X_{2}$. As the amount of overlap increases, the redundant information increases. The distance function used was the $L_{1}$ norm of the difference. (**C**) Per class redundant information for different channels, crops, and frequency decompositions, with β=50 used in the optimization.

We analyze the redundant information between different views of the same CIFAR-10 image (Figure 2) by optimizing over a model family of ResNet-18’s [15], described in Appendix C.6. In particular, we split the image in two crops, a left crop $X_{1}$ containing all pixels in the first *w* columns, and a right crop $X_{2}$ containing all pixels in the last *w* columns (Figure A3). Intuitively, we expect that as the width of the crop *w* increases, the two views will overlap more, and the redundant information that they have about the task will increase. Indeed, this is what we observe in Figure 2B.

We next study the redundant information between different sensor modalities. In particular, we decompose the images into different color channels ($X_{1}=\mathrm{red}\;\mathrm{channel}$ and $X_{2}=\mathrm{blue}\;\mathrm{channel}$), and frequencies ($X_{1}$ = high-pass filter and $X_{2}$ = low-pass filter). We show example images in Figure A3. As expected, different color channels have highly redundant information about the task (Figure 2C), except when discriminating classes (such as dogs and cats) where precise color information (coming from using jointly the two channels synergistically) may prove useful. On the contrary, the high-frequency and low-frequency spectrum of the image has a lower amount of redundant information, which is also expected since the high and low-frequencies carry complementary information. We also observe that the left and right crops of the image are more redundant for pictures of cars than other classes. This is consistent with the fact that many images of cars in CIFAR-10 are symmetric frontal pictures of cars, and can easily be classified using just half of the image. Overall, there is more redundant information between channels, then crops, then frequencies. Together, these results show that we can compute the redundant information of high dimensional sources, providing empirical validation for our approximation and a scalable approach to apply in other domains.

### 4.3. Neural Data Decoding

**Figure 3:**
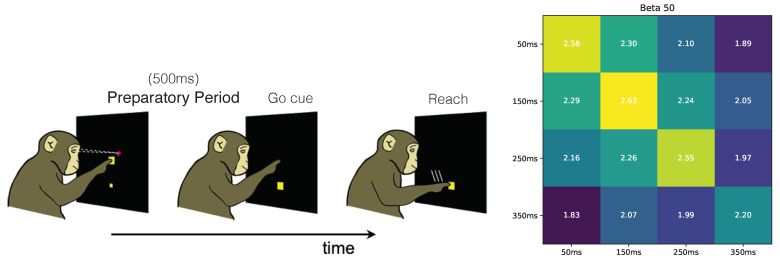
(**Left**): Schematic of delayed-center-out reaching task. There are 8 possible target locations (equally spaced), one of which is shown. Neural data are recorded from the premotor cortex of a monkey, using 97 electrodes. (**Right**): Redundant information between short disjoint time windows during the preparatory period, before a reach can be initiated. Even before the reach is initiated, the target location can be decoded from the premotor cortex, using neural data averaged in a short 100 ms time window. In the confusion matrix, adjacent time bins have higher redundant information about the target location during the preparatory period, reflecting that the encoding of the target location is more similar in adjacent time windows.

We next applied our framework to analyze how information is encoded in motor-related cortical regions of monkeys during the preparatory period of a center-out reaching task [17]. Our goal was to confirm prior hypotheses known about motor cortical encoding from the literature. In the center-out reaching task, there are 8 target locations and the monkey needs to make a reach to one of the targets depending on a cue (Figure 3 Left). Our data set consists of a population recording of spike trains from 97 neurons in the dorsal premotor cortex (PMd) during trials that were 700 ms long. Each trial comprises a 200 ms baseline period (before the reach target is turned on) and a 500 ms preparatory (planning) period after the reach target is turned on but before the monkey can initiate a reach. Both our training and testing data sets consisted of 91 reaches to each target. During the 500 ms preparatory period, the monkey prepared to reach toward a target but did not initiate the reach, enabling us to study the PMd neural representation of the planned reach to the target.

First, we used RINE to compute redundant information of the PMd activity over time during the delay period. PMd activity is known to be relatively static during the delay period, approaching a stable attractor state [18]. We therefore expect the redundant information between adjacent time windows to be high. To quantify this, we evaluated the redundant information between different time segments of length 100 ms, beginning 50 ms after the beginning of the preparatory period. For our feature vector, we counted the total number of spikes for each neuron during the time segment. We note that even in the relatively short window of 100 ms, there is a significant amount of usable information about the target in the recorded population of neurons, since the diagonal elements of Figure 3 are close to 3 bits. This is consistent with prior studies that show that small windows of preparatory activity can be used to decode the target identity [17,19]. We also found that adjacent time windows contain higher redundant information (closer to the 3 bits), consistent with the idea that the encoding of the target between adjacent time windows are more similar [20]. Together, these results show that RINE computes redundant information values consistent with results reported in the literature showing that PMd representations stably encode a planned target.

**Figure 4:**
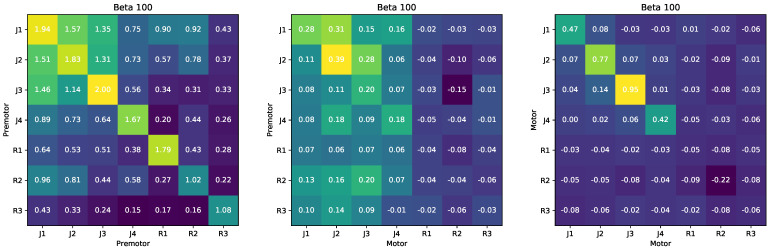
Neural decoding confusion matrix for different monkeys and different sessions (**Left**), motor and premotor cortex (**Middle**) and between motor cortex across different monkeys and sessions (**Right**).

Second, we used RINE to study the redundant information between the neural activity recorded on different days and between subjects. We analyzed data from another delayed-center-out task with 8 targets and a variable 400–800 ms delay period, during which the monkey could prepare to reach to the target, but was not allowed to initiate the reach (Appendix C.7.2). We examined the redundant information about the target location in the premotor cortex on different sessions and between the different monkeys, Monkey J and Monkey R. When data came from different sessions, we generated a surrogate data set by conditioning on the desired target reach, ensuring that $X_{1}$ and $X_{2}$ corresponded to the same target *Y*. At an extreme, if we could only decode 4 of the 8 targets from Monkey J’s PMd activity and the other 4 of the 8 targets from Monkey R’s PMd activity, there would be no redundant information in the recorded PMd activity. Our results are shown in Figure 4 Left. Since the PMd electrodes randomly sample tens of neurons out of hundreds of millions in the motor cortex, we expect the redundant information between Monkey J and Monkey R PMd recordings to be relatively low. We also expect the redundant information across sessions for the same monkey to be higher since the electrodes are relatively stable across days [21]. RINE calculations are consistent with these prior expectations. We found that the redundant information is higher between sessions recorded from the same monkey than between sessions recorded from different monkeys.

Finally, we quantified redundant information between PMd and the primary motor cortex (M1) during the delay period (Figure 4 Middle). We expect redundant information to be relatively low; whereas PMd strongly represents the motor plan through an attractor state, activity in M1 is more strongly implicated in generating movements with dynamic activity [22]. We find that the values of the redundant information between PMd and M1 are low (0.4 to 0.7 bits), indicating that there is little redundant encoding of target information during the delay period between premotor and motor cortex, even for the same monkey. This is consistent with these two regions having distinct roles related to the initiation and execution of movement [18]. One explanation for having low redundant information between the motor and the premotor cortex during the preparatory period is that there is little encoding of the target location in the motor cortex during the preparatory period, and that the motor cortex serves a role more related to producing appropriate muscle activity. Similar to how we analyzed the redundant information between the premotor cortex, we analyzed the redundant information between the motor cortex across sessions (Figure 4 Right). We find that there is little information about the planned target in M1 activity for both monkeys (far from 3 bits). Monkey R’s M1 information is particularly low due to M1 electrodes recording from very few neurons. The lower values of redundant information between motor cortices compared to premotor cortices implies that there is less information in M1 than PMd about the target during the preparatory, consistent with prior literature.

### 4.4. Advantage of Redundant Information as a Task-Related Similarity Measure

How does the notion of redundancy compare to other similarity metrics such as $I(X_{1};X_{2})$ or the cosine similarity between $X_{1}$ and $X_{2}$? Critically, both measures are agnostic to a target *Y*, whereas the redundant information reflects the common information about the target *Y*. Hence, the redundant information is unaffected by factors of variation that are either pure noise or caused by target-independent factors, but these factors of variation affect other similarity metrics. This may be particularly important in neuroscience, since recordings from different areas or neurons contain significant noise or non-task variability that can affect similarity metrics. We designed a synthetic task to showcase these effects. The task is similar to the neural center-out reaching task, with 8 classes. The task was designed so that each input $X_{1}$ and $X_{2}$ contains information about *n* classes, with the minimum overlap between the classes specified: when each input specifies n=4 classes, there are no classes that are encoded by both $X_{1}$ and $X_{2}$ (hence 0 bits of redundant information), and with n=5 classes it means that 2 common classes are encoded by the two inputs.

**Figure 5:**
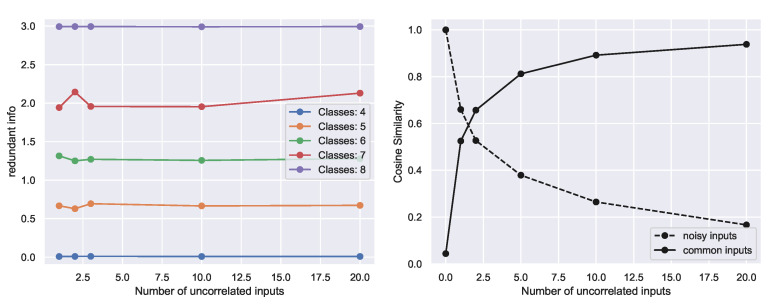
Comparison of redundant information against cosine similarity metric. (**Left**) The redundant information is invariant to the number of uncorrelated inputs, and we validate empirically that our approximation of redundant information remains approximately constant with increasing number of uncorrelated inputs. (**Right**) In contrast, alternative similarity metrics, such as the cosine similarity, decreases with increasing number of random noisy units (dashed lines) or increases with correlated non-task units, (solid line).

In Figure 5 Left, we show that the redundant information increases with increasing the overlap between the classes specified by the input, but the redundant information is unaffected by adding units that are uncorrelated with the target, evidenced by approximately flat lines for each value of *n*. In contrast, the cosine similarity is affected by the addition of such units (Figure 5 Right). Adding noisy inputs decreases the cosine similarity, whereas the addition of shared non-task-related inputs increases the cosine similarity (Appendix C.5). Thus, the important distinction of the redundant information in comparison to direct similarity metrics applied on the inputs is that the redundant information captures information in sources about a *target Y*, whereas direct similarity metrics applied on the sources are agnostic to the target or task *Y*.

## 5. Discussion

Central to the Partial Information Decomposition, the notion of redundant information offers promise for characterizing the component of task-related information present across a set of sources. Despite its appeal for providing a more fine-grained depiction of the information content of multiple sources, it has proven difficult to compute in high-dimensions, limiting widespread adoption. Here, we show that existing definitions of redundancy can be recast in terms of optimization over a family of deterministic or stochastic functions. By optimizing over a subset of these functions, we show empirically that we can recover the redundant information on simple benchmark tasks and that we can indeed approximate the redundant information for high-dimensional predictors.

**Figure A2:**
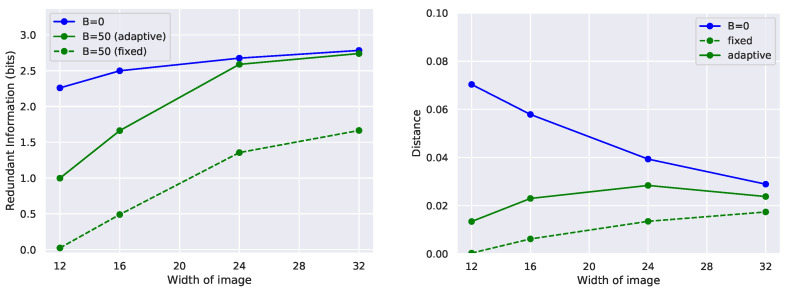
(**Left**) If B=50 for all epochs of training, the network is stuck in a trivial solution in learning. Setting β adaptively leads to an improved solution. (**Right**) The final distance terms are comparable.

**Figure A3:**
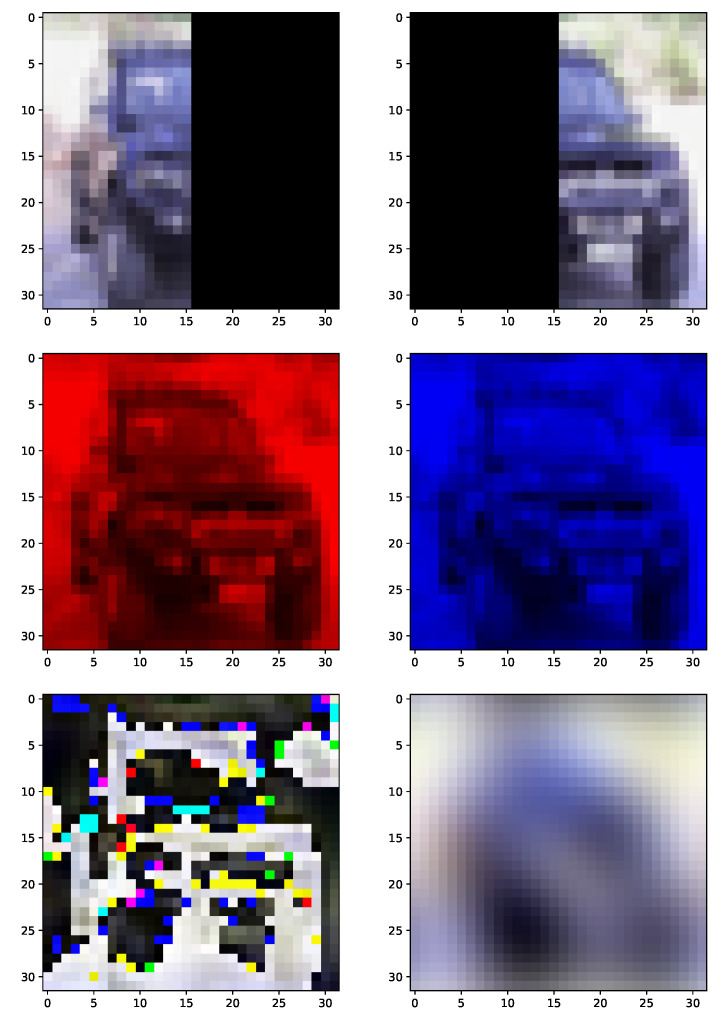
Example decompositions of an image (car) from CIFAR-10. This is an example of $x_{1}$ and $x_{2}$ in our CIFAR experiments. **Top**: different crops; **Middle**: colors of channels; and **Bottom**: frequencies.

Although our approach correctly computes the redundant information on canonical examples as well as provides intuitive values on higher-dimensional examples when ground-truth values are unavailable, with all optimization of overparametrized networks on a finite training set, there is the possibility of overfitting to features in the training set and having poor generalization on the test set. This is not just a problem for our method, but is a general feature of many deep learning systems, and it is common to use regularization to help mitigate this. PAC-style bounds on the test set risk that factor in the finite nature of the training set exist [23], and it would be interesting to derive similar bounds that could be applied on the distance term to bound the deviation on the test set. Additionally, future work should investigate the properties arising from the choice of distance term since other distance terms could have preferable optimization properties or desirable information-theoretic interpretations, especially when it is non-zero. Last, the choice of beta-schedule beginning with a small value and increasing during training was important (Figure A2), and may need to be tuned to a particular task.

Our approach only provides a value summarizing how much of the information in a set of sources is redundant, and it does not detail what aspects of the sources are redundant. For instance, when computing the redundant information in the image classification tasks, we optimized over a high-dimensional parameter space, learning a complicated nonlinear function. Although we know the exact function mapping the input sources to prediction, it is difficult to identify the “features” or aspects of the input that contributed most to the prediction. Future work should try to extend our work to describe not only how much information is redundant, but what parts of the sources are redundant.

## Figures and Tables

**Table 1:** Comparison of redundancy measures on canonical examples. Quantities are in bits, and $I_{\cap }^{V}$ denotes our approximation, shown in bold (for β=15). The mean and standard deviation (inside parentheses) are reported over 5 different initializations. $I_{\cap }^{\wedge }$ denotes the redundant information in Griffith et al. [2] and $I_{\cap }^{\mathrm{GH}}$ denotes the redundant information in Griffith and Ho [5]. Note that Kolchinsky [7] computed $I_{\cap }^{\mathrm{GH}}$ for the AND operation and obtained 0.123 bits, as opposed to the 0 bits reported in [5]. We carry out this computation for different values of β in Table A5.

	True	$I_{\cap }^{\wedge }$	$I_{\cap }^{\mathrm{GH}}$	$I_{\cap }^{V}$ (β=15)
UNQ [Table A1]	0	0	0	**0.006 (0.016)**
AND [Table A2]	[0, 0.311]	0	0	**0.007 (0.001)**
RDNXOR [Table A3]	1	1	1	**0.977 (9 × 10^−4^)**
IMPERFECTRDN [Table A4]	0.99	0	0.99	**0.984 (0.002)**

## Data Availability

Code is available at: www.github.com/mjkleinman/RINE.

## Appendix A. Canonical Tasks

The probabilities on the right hand side of the table denote the probability $p(x_{1},x_{2},y)$.

**Table A1:** UNQ: $X_{1}$ and $X_{2}$ contribute uniquely 1 bit of Y. Hence, there is no redundant and synergistic information.

$X_{1}$	$X_{2}$	*Y*	
a	b	ab	1/4
a	B	aB	1/4
A	b	Ab	1/4
A	B	AB	1/4

**Table A2:** AND: $X_{1}$ and $X_{2}$ combine nonlinearly to produce the output *Y*. It is generally accepted that the redundant information is between [0, 0.311] bits [5], where $I\left(X_{1};Y\right)=I\left(X_{2};Y\right)=0.311$ bits.

$X_{1}$	$X_{2}$	*Y*	
0	0	0	1/4
0	1	0	1/4
1	0	0	1/4
1	1	1	1/4

**Table A3:** RDNXOR: A combination of redundant a synergistic information where $X_{1}$ and $X_{2}$ contributes 1 bit of redundant information, and 1 bit of synergistic information.

$X_{1}$	$X_{2}$	*Y*	
r0	r0	r0	1/8
r0	r1	r1	1/8
r1	r0	r1	1/8
r1	r1	r0	1/8
R0	R0	R0	1/8
R0	R1	R1	1/8
R1	R0	R1	1/8
R1	R1	R0	1/8

**Table A4:** IMPERFECTRDN: $X_{1}$ fully specifies the output, with $X_{2}$ having a small chance of flipping one of the bits. There should be 0.99 bits of redundant information.

$X_{1}$	$X_{2}$	*Y*	
0	0	0	0.499
0	1	0	0.001
1	1	1	0.500

## Appendix B. Proofs

**Proposition** **A1.**
*Let V={f:X→P(Y)} consist of the family of deterministic functions from X to distributions over Y. Then $I_{\cap }^{V}=I_{\cap }^{\wedge }$.*

**Proof.** We show that $I_{\cap }^{V}=I_{\cap }^{\wedge }$ by proving both inequalities $I_{\cap }^{V}\geq I_{\cap }^{\wedge }$ and $I_{\cap }^{V}\leq I_{\cap }^{\wedge }$. To show that $I_{\cap }^{V}\geq I_{\cap }^{\wedge }$. Let $f_{i}:X\to Q$ be the functions that maximize Equation (2), and let $Q=f_{i}\left(X_{i}\right)$. Let p(y|q) be the corresponding optimal decoder. Define ${\hat{f}}_{i}:X\to P\left(Y\right)$ as ${\hat{f}}_{i}\left(x\right)=p\left(y|f_{i}\left(x\right)\right)$. Note that

$$\begin{array}{cc} H_{{\hat{f}}_{i}}\left(Y|X_{i}\right) & =-\int p\left(y,x\right)\log p(y|f_{i}\left(x\right))dx\;dy\\ \; & =-\int p\left(y,x\right)\left(\int \delta_{q,f_{i}\left(x\right)}\log p\left(y|q\right)dq\right)dx\;dy\\ \; & =-\int p\left(y,x\right)\left(\int p(q|x,y)\log p(y|q)dq\right)dx\;dy\\ \; & =-\int p(q,x,y)\log p(y|q)dq\;dx\;dy\\ \; & =-\int p(q,y)\log p(y|q)dq\;dy\\ \; & =H(Y|Q), \end{array}$$

where between the first and second line we used the definition of dirac delta; between the second and third we used the definition of $p\left(q|x\right)=\delta_{q,f_{i}\left(x\right)}$; and between the fourth and fifth line we marginalized over *x*. Using this result in Equations (6) and (8), we obtain the following:

$$I_{\cap }^{V}\geq H\left(Y\right)-H\left(Y|Q\right)=I\left(Y;Q\right)=I_{\cap }^{\wedge }.$$

The above inequality is obtained because ${\hat{f}}_{i}\in V=U$ but is not necessarily the function corresponding to the infimum. To show that $I_{\cap }^{V}\leq I_{\cap }^{\wedge }$, let $f_{i}:X\to Q$ and let $Q=f_{i}\left(X_{i}\right)$. Define ${\hat{f}}_{i}:X\to P\left(Y\right)$ as ${\hat{f}}_{i}\left(x\right)=\hat{p}\left(y|f_{i}\left(x\right)\right)$ where ${\hat{f}}_{i}$ satisfies Equations (6) and (7). Note that

$$\begin{array}{cc} I_{\cap }^{V} & =H\left(Y\right)-H_{\hat{f_{i}}}\left(Y|X\right)\\ \; & =H(Y)-H(Y|Q)\\ \; & =I(Y;Q)\\ \; & \leq I_{\cap }^{\wedge }. \end{array}$$

The second equality comes since we showed above $H\left(Y|Q\right)=H_{\hat{f_{i}}}\left(Y|X\right)$. The inequality comes since *Q* satisfies the constraint of Equation (2) but does not necessarily maximize the objective. □

**Proposition** **A2.**
*Let t:X→X be any invertible transformation in V. Then:*

(A1) $$I_{\cap }^{V}\left(X_{1};X_{2}\;\to \;Y\right)=I_{\cap }^{V}\left(t_{1}\left(X_{1}\right);t_{2}\left(X_{2}\right)\;\to \;Y\right)$$

**Proof.** We define an invertible transformation in V to be one such that f∘t∈V for all f∈V, which implies that $f\circ t^{-1}\in V$. Recall that $I_{\cap }^{V}:=H\left(Y\right)-L_{\cap }^{V}$ (Equation (8)), and note that H(Y) is not affected by transformations on the sources. Let $L^{*}$ correspond to the minimum of the following:

(A2) $$\min_{f_{1},f_{2}\in V}\frac{1}{2}\left[H_{f_{1}}\left(Y|X_{1}\right)+H_{f_{2}}\left(Y|X_{2}\right)\right]\;s.t\;D(f_{1},f_{2})=0.$$

And let $L_{t}^{*}$ correspond to the minimum of

(A3) $$\min_{{\tilde{f}}_{1},{\tilde{f}}_{2}\in V}\frac{1}{2}\left[H_{{\tilde{f}}_{1}}\left(Y|t_{1}\left(X_{1}\right)\right)+H_{{\tilde{f}}_{2}}\left(Y|t_{2}\left(X_{2}\right)\right)\right]\;s.t\;D({\tilde{f}}_{1},{\tilde{f}}_{2})=0.$$

We will show that $L^{*}=L_{t}^{*}$. Let

$${\tilde{f}}_{1}=f_{1}\circ t_{1}^{-1}\in V,$$

$${\tilde{f}}_{2}=f_{2}^{}\circ t_{2}^{-1}\in V,$$

where ${\tilde{f}}_{1},{\tilde{f}}_{2},f_{1}^{},f_{2}^{}\in V\subseteq U=\left\{f:X\to P\left(Y\right)\right\}$. We can rewrite Equation (A3) by canceling out $t^{-1}\circ t$ as shown below so that:

$$\begin{array}{cc} L_{t}^{*} & =\min_{{\tilde{f}}_{1}^{},{\tilde{f}}_{2}^{}\in V}\frac{1}{2}\left[H_{{\tilde{f}}_{1}^{}}\left(Y|t_{1}\left(X_{1}\right)\right)+H_{{\tilde{f}}_{2}^{}}\left(Y|t_{2}\left(X_{2}\right)\right)\right]\;s.t\;D({\tilde{f}}_{1}^{},{\tilde{f}}_{2}^{})=0\\ \; & =\min_{f_{1}^{\circ }t_{1}^{-1},f_{2}^{\circ }t_{2}^{-1}\in V}\frac{1}{2}\left[H_{f_{1}\circ t_{1}^{-1}}\left(Y|t_{1}\left(X_{1}\right)\right)+H_{f_{2}\circ t_{2}^{-1}}\left(Y|t_{2}\left(X_{2}\right)\right)\right]\;s.t\;D(f_{1}\circ t_{1}^{-1},f_{2}\circ t_{2}^{-1})=0\\ \; & =\min_{f_{1},f_{2}\in V}\frac{1}{2}\left[H_{f_{1}}\left(Y|X_{1}\right)+H_{f_{2}}\left(Y|X_{2}\right)\right]\;s.t\;D(f_{1},f_{2})=0\\ \; & =L^{*}. \end{array}$$

□

## Appendix C. Additional Details

### Appendix C.1. Partial Information Decomposition

Information theory provides a principled framework for understanding the dependencies of random variables through the notion of mutual information [24]. However, information theory does not naturally describe how the information about a target *Y* is distributed among a set of sources $X_{1},\ldots X_{n}$. For example, ideally, we could decompose the mutual information $I(X_{1},X_{2};Y)$ into a set of constituents describing how much information that $X_{1}$ contained about *Y* was also contained in $X_{2}$, how much information about *Y* was unique to $X_{1}$ (or $X_{2}$), as well as how much information about *Y* was only present when knowing both $X_{1}$ and $X_{2}$ together. These ideas were introduced in Williams and Beer [1] as the Partial Information Decomposition (PID).

Standard information-theoretic quantities $I(X_{1};Y)$, $I(X_{1};Y|X_{2})$, and $I(X_{1},X_{2};Y)$ can be formed with components of the decomposition:

(A4) $$I\left(X_{1};Y\right)=UI\left(X_{1};Y\right)+I_{\cap }$$

(A5) $$I\left(X_{2};Y|X_{1}\right)=UI\left(X_{2};Y\right)+SI$$

(A6) $$I\left(X_{1},X_{2};Y\right)=UI\left(X_{1};Y\right)+SI+UI\left(X_{2};Y\right)+I_{\cap }$$

Here, UI represents the “unique” information and SI represents the “synergistic” information. Equation (A6) comes from the chain rule of mutual information, and by combining Equations (A4) and (A5). These quantities are shown in the PID diagram shown in Figure A1. Computing any of these quantities allows us to compute all of them [3]. In Banerjee et al. [6], they described an approach to compute the unique information, which was only feasible in low dimensions. In our paper, we instead focus on computing the “redundant” information.

### Appendix C.2. Alternative Notion of Redundancy

Recently Kolchinsky [7] proposed to quantify redundancy through the following optimization problem:

(A7) $$I_{\cap }^{K}\left(X_{1};\ldots ;X_{n}\;\to \;Y\right):=\max_{s_{Q|Y}}\;I\left(Q;Y\right)\;s.t.\;\forall i\;s_{Q|Y}⪯p_{X_{i}|Y}$$

The notation $s_{Q|Y}⪯p_{X_{i}|Y}$ indicates that there exists a channel $p_{Q|X_{i}}$ such that Equation (A8) holds for all *q* and *y*.

(A8) $$s\left(q|y\right)=\sum_{x_{i}}p\left(q|x_{i}\right)p\left(x_{i}|y\right).$$

In a sense, Equation (A8) indicates that *Q* is a “statistic” of $X_{i}$.

### Appendix C.3. Setting Value of β

When optimizing the equation in practice, it is more difficult to optimize initially using very large values of β since the network could easily learn a trivial solution. We therefore adaptively set β depending on the epoch of training. In this manner, we find that the network settles in a redundant solution that performs well on the task, as opposed to a solution that is trivial. We smoothly increase $\beta_{i}$ during training following the formula so that the value of β at epoch *i* is (γ = 0.97):

(A9) $$\beta_{i}=\beta \left(1-\gamma^{i}\right).$$

We also perform an ablation study where we fix $\beta_{i}=\beta$, and find that the network settles at a more trivial solution (Figure A2).

### Appendix C.4. Training Details for Canonical Examples

We trained a small fully-connected network with hidden layers of size [25−15−10], using batch normalization and ReLU activations, with an initial learning rate of 0.01 decaying smoothly by 0.97 per epoch, for 30 epochs. We generated a data set consisting of 10,000 samples of which 80% corresponded to training data, and the remaining 20% corresponded to the test data. We trained with different values of β. β=0 corresponds to the average usable information of $I_{u}\left(X_{1};Y\right)$ and $I_{u}\left(X_{2};Y\right)$. As β increases, the quantity $I_{\cap }^{V}$ more strongly reflects redundant information. RINE produces values close to the ground truth for these canonical examples. The tasks, with their corresponding inputs, outputs and associated probabilities are shown in Appendix A. Our comparison is shown in Table 1. Note that there is some randomness that occurs due to different initialization optimizing the neural networks; hence, the values may differ slightly.

### Appendix C.5. Comparison with Cosine Similarity

To highlight the difference between the redundant information that two inputs $X_{1}$ and $X_{2}$ have about a task *Y* and a direct similarity measure that could be applied on $X_{1}$ and $X_{2}$, we designed a synthetic task. In this task, there are 8 classes. We designed the inputs so that each input $X_{1}$ and $X_{2}$ would contain information about *n* classes, with minimal overlap. For instance, if n=4, each input would contain information about 4 distinct classes, so there would be no redundant information. We swept the value of *n* ranging from 4 to 8 (Figure 5 Left, with increasing redundant information for increasing values of *n*). We optimized over a two-hidden-layer deterministic neural network with hidden layer dimensions of 25 and 15, using Adam with a learning rate of 0.005 for 50 epoch, with β=50. We added noisy inputs with each input coming from $N(0,2^{2})$. These inputs did not affect the value of redundant information; however, adding noisy inputs decreases the cosine similarity (shown for the case of n=8), whereas the addition of non-task related common inputs increases the cosine similarity (shown for the case of n=4).

### Appendix C.6. Training Details for CIFAR-10

To compute the redundant information for CIFAR-10, we optimized over the weights in Equation (6) using ResNet-18’s [15]. We trained the network for 40 epochs, with an initial learning rate of 0.075, decreasing smoothly by 0.97 per epoch, with weight decay of 0.005. We show example images that represent the inputs $x_{1}$ and $x_{2}$ in Figure A3. We jointly trained two networks that process inputs $x_{1}$ and $x_{2}$, respectively, constrained to have similar predictions through including $D(f_{1},f_{2})$ in the loss. To compute $D(f_{1},f_{2})$, we quantified the $L_{1}$ norm of the distance between the softmax scores of the predictions. We evaluated the cross-entropy loss on the test set.

### Appendix C.7. Training Details for Neural Decoding

#### Appendix C.7.1. Fixed Delay Center Out Task

In this task, there are 8 target locations. After a target is shown, the monkey makes a plan to reach towards the target. The monkey then reaches to the target after a go cue (Figure 3 Left). Our data set consisted of a population recording of spike trains from 97 neurons in the premotor cortex during trials that were 700 ms long. Each trial comprises a 200 ms baseline period (before the reach target turned on) and a 500 ms preparatory (planning) period after the reach target turned on but before the monkey can initiate a reach. Both our training and testing data sets consisted of 91 reaches to each target.

#### Appendix C.7.2. Variable Delay Center Out Task

We analyzed data from another delayed-center-out task with 8 targets with a variable 400–800 ms delay period, during which the monkey could prepare to reach to the target, but was not allowed to initiate the reach until the go cue. In these data sets, there were significantly fewer total trials per session (220 total reaches across 8 targets) in comparison to the data set with a fixed delay period. Data from two motor-related regions, the premotor and primary motor cortex, was recorded from 2 monkeys (J and R). There were 4 sessions associated with monkey J and 3 sessions associated with monkey *R*. We used 90% of the trials to train and 10% of the trials to test, and the plots reflect the redundant information on the test set.

### Appendix C.8. Generalization to n Sources

Our formulation naturally generalizes to *n* sources $X_{1},\ldots ,X_{n}$. In particular, Equation (9) can be generalized as follows:

(A10) $$L_{\cap }^{V}\left(X_{1};\ldots ;X_{N}\;\to \;Y,\beta \right):=\min_{f_{1},\ldots ,f_{n}\in V}\frac{1}{n}\left[\sum_{i=1}^{n}H_{f_{i}}\left(Y|X_{i}\right)\right]+\beta D\left(f_{1},\ldots ,f_{n}\right).$$

We note that when computing the redundant information, we compute the loss without the distance term $D(f_{1},\ldots ,f_{n})$. A naive extension of the distance term to *n* sources is computing the sum of all the pairwise distance terms. If the number of sources is large, however, it may be beneficial to consider efficient approximations of this distance term.

### Appendix C.9. Details on Canonical Examples

**Table A5:** Comparison of redundancy measures on canonical examples for additional values of β than Table 1. Quantities are in bits. $I_{\cap }^{V}$ denotes our variational approximation for different values of β. $I_{\cap }^{\wedge }$ denotes the redundant information in Griffith et al. [2] and $I_{\cap }^{\mathrm{GH}}$ corresponds to the redundant information in Griffith and Ho [5].

	True	$I_{\cap }^{\wedge }$	$I_{\cap }^{\mathrm{GH}}$	$I_{\cap }^{V}$ (β=0)	$I_{\cap }^{V}$ (β=5)	$I_{\cap }^{V}$ (β=15)
UNQ [Table A1]	0	0	0	0.981	0.809	0.006
AND [Table A2]	[0, 0.311]	0	0	0.318	0.008	0.007
RDNXOR [Table A3]	1	1	1	0.981	0.983	0.977
IMPERFECTRDN [Table A4]	0.99	0	0.99	0.983	0.978	0.984
